# Scoliosis: lower limb asymmetries during the gait cycle

**DOI:** 10.1186/s40945-015-0001-1

**Published:** 2015-07-08

**Authors:** Cassandra Kay Haber, Mark Sacco

**Affiliations:** 1 Department of Physiotherapy, Mater Dei Hospital, Msida, Malta; 2 Department of Physiotherapy, Faculty of Health Sciences, Mater Dei Hospital, Room 10, Block A, Level 1, Msida, Malta

**Keywords:** Scoliosis, Gait analysis, Speed of gait, Ground reaction force, Electromyography

## Abstract

**Background:**

Several studies indicate that the gait pattern of subjects suffering from scoliosis differs from the norm. However, there is conflicting evidence regarding the source of this discrepancy.

**Objective:**

To evaluate lower limb asymmetries in selected gait variables.

**Study design:**

A case–control study on lower limb asymmetries during gait which can be related to scoliosis.

**Methods:**

31 subjects with scoliosis (Study Group - SG) and an equal comparative control sample (Control Group – CG) of subjects underwent objective gait analysis with the Vicon® motion caption system whilst walking at a comfortable speed along the gait laboratory walkway. Analysis was performed at three levels: (1) Asymmetry in the SG against asymmetry in the CG, (2) Difference in magnitude of asymmetry between the SG and CG, and (3) Global mean values in the SG vs. CG. The Paired Student *T*-Test was used for intra-group analysis whilst the Independent Student *T*-Test was used for inter-group analysis of the selected parameters, which include temporal parameters (stride length, stride time, step length, individual step speed, speed of gait, cadence, swing-to-stance ratio), ground reaction force (peak GRF values during Loading and Propulsion phases, vertical component only) and electromyography (peak EMG values and their time of onset, as a percentage of the gait cycle) of two lower limb muscles (Gastronemius and Vastus Medialis).

**Results:**

No intra-group variation was found to be significant. However, the speed of gait was found to be significantly slower (*p* = 0.03) in scoliotic subjects when compared to the norm, as a result of the shorter stride length (*p* = 0.002 and longer stride time (*p* = 0.001) in the SG. Furthermore, there was statistical significance in the time of onset of EMG peaks for the Lateral Gastrocnemius (*p* = 0.02) with regards to inter-group difference in magnitude of lower limb asymmetry and global mean values.

**Conclusions:**

Scoliosis is a tri-planar deformity which has some impact on the gait pattern. This research study concludes that scoliotic subjects have a slower speed of gait due to a shorter stride length and a longer stride time, together with variations in the timing of muscle activation.

## Background

Scoliosis is a spinal deformity occurring in the frontal, sagittal and transverse planes with multiple variations in presentation. This deformity generates postural changes [1], sensory disturbance [1, 2], standing instability [1, 3, 4] as well as gait pattern modifications. Gait is the most common of all human movements and it can be defined as a harmonious, energetically efficient activity resulting from a sequence of alternate lower limb steps [5]. Although scoliosis is located in the spine, present literature suggests that the step length [6–9], cadence [10] and velocity [11], hip, knee and ankle range of motion [7–9,12–14], loading and unloading [15], duration of trunk and gluteal muscle activation [6, 7], magnitude of muscle force [15,16], energy cost and muscle efficiency [7–9, 11] are affected. However, there is conflicting evidence regarding the effect of the condition on left and right lower limb asymmetries [3, 7, 15–17].

There are several studies which analyse the asymmetries in muscles of the back [18, 19]. However, few studies have focussed on lower limb asymmetries and the need for further research has been voiced by several authors [4, 7, 8, 15]. A comprehensive evaluation of lower limb asymmetries during the gait of individuals suffering from scoliosis may help one appreciate the physical implications of this condition. Therefore, the aim of this research is to analyse the effect of Idiopathic Scoliosis on gait, through kinematic, kinetic and electromyographic (EMG) analysis. Left and right lower limb asymmetries in EMG values, vertical ground reaction force (GRF) and temporal parameters will be evaluated in relation to the side of spinal curvature occurring in scoliosis, taking into consideration the concavity versus the convexity aspect relative to the erect vertical spine. The purpose of unifying these variables is to obtain a generalised understanding of gait with special emphasis on lower limb asymmetries, which may not be evident unless sophisticated gait analysis technology is utilised [7, 15]. Differences and correlations will be estimated between the variables of the Study Group (SG) and the norm via a comparison Control Group (CG) of non-scoliosis individuals.

In summary, it is hypothesised that (1) the direction of the scoliotic curve will result in an asymmetrical gait pattern, (2) the extent of the asymmetry will be different for the SG and the CG, and (3) there will be a difference between the gait pattern of the SG and the CG.

## Materials and methods

### Participants

This case–control study follows a quantitative comparative research design, consisting of a total of 62 subjects: 31 scoliotic subjects in the SG and 31 non-scoliotic subjects in the CG. Data collection took place at the Biomedical Engineering Laboratory at the University of Malta in the beginning of the year 2013. The subjects for the SG were selected from individuals with a recorded medical diagnosis of idiopathic scoliosis registered in the database of a Maltese public hospital and referred to the Physiotherapy Department during the year 2012. All individuals who met the inclusion criteria set for the study (Table 1) were invited to participate. Thus, the size of the study group was determined by voluntary participation of the scoliotic individuals, consent of the legal guardians in the case of children, and the availability of the gait laboratory. An appropriate CG of subjects with no spinal deformity, matched by age and gender to the study subjects, was acquired through random sampling from a school. The study was approved by the University Research Ethics Committee and the Data Protection Officer prior to commencement, and all ethical rules of conduct (including informed consent from all participants) were abided by throughout the whole research process.

**Table 1:** The inclusion criteria of the research population

Study group	Control group
Diagnosis of Idiopathic Scoliosis	No diagnosis of scoliosis
No surgical correction of the spinal deformity	Male or female
	Age: 10–30 years
Male or female	Scoliometric reading of less than 2
Age: 10–30 years	
Scoliometric reading of at least 5°	Symmetrical leg length
Leg length discrepancy (LLD) less than 2 cm	No relevant orthopaedic condition
No relevant orthopaedic condition	No concomitant diseases present which could influence the gait pattern
No concomitant diseases present which could influence the gait pattern	Voluntary participation
Voluntary participation	

### Procedure

Prior to gait analysis, the severity of the scoliosis was determined via Scoliometer® measurements taken with the subject in the Forward Bend Test position in standing. Scoliometric readings were taken three times and the best readings were recorded. Subjects were only admitted in the SG if they presented with Scoliometric reading of at least 5°, since lower curvatures are associated with non-scoliotic spines [20,21]. The objective gait analysis was performed using the Vicon® motion capture system at a gait laboratory, which consists of: a 10 m walkway, six video cameras with infrared circular strobes, EMGs, and a force plate (AMTI) controlled by the Vicon Nexus^TM^ Software. The Lower Body Plug-in Gait marker set, which describes the lower half of the biomechanical model and requires a total of sixteen markers (four on the pelvis and six on each lower limb), was adopted for this study. Marker data was collected at 100Hz, whilst force plate and EMG data was collected at 1000Hz simultaneously. EMG data was rectified and smoothened, but not normalised to MVC since peak values were one of the analysed variables. ‘Surface Electromyography for the Non-Invasive Assessment of Muscles’ (SENIAM) recommendations for the electrode size, electrode material, sensor construction and electrode placement were applied. The surface electrode location relative to the target muscle was accurately determined by a palpable contraction during a low effort muscle test. In this study, the muscles chosen for investigation by the researcher were Vastus Medialis (VM) and both lateral (GL) and medial (GM) heads of Gastrocnemius.

Once all preparations were complete, the subject was asked to walk a number of times along the walkway at a comfortable speed. Ten trials were collected with the right foot landing on a concealed force plate, and another ten trials with the left foot landing on the force plate. Synchronisation of the EMG signals and force plate signals with the events of the gait cycle, namely Initial Contact and Toe Off, was performed via the Vicon Nexus^TM^ Software. This allowed better analysis of the muscle action with respect to the gait cycle.

### Data analysis

Data was extracted from the Vicon Nexus^TM^ and Vicon Polygon^TM^ Software into Microsoft Excel® in order to analyse lower limb discrepancies in temporal parameters (stride length, stride time, step length, individual step speed, speed of gait, cadence and swing-to-stance ratio), the peak vertical GRF values during the Loading and the Propulsion phase, and the peak EMG value and the time it occurs as a percentage of the whole gait cycle. Since peak muscle activity of the selected muscles in this study occur during the stance phase, the time of onset of peak EMG values was analysed at the time after Initial Contact and the time before Toe Off. All parameters were checked for normal distribution using the Shapiro-Wilk test. Mean values and standard deviations were calculated.

During intra-group analysis, data arising from the lower limbs related to the concavity side and the convexity side of the scoliotic curve in the SG were assumed independent and treated as separate data points. In the CG, the left and right lower limbs were considered. Significant differences between values were obtained from the Paired Student *T*-Test at a 95 % confidence level for intra-group analysis.

During inter-group analysis, mean values and side-to-side lower limb asymmetry values were analysed for each mentioned parameter using the Independent *T*-Test, also at a 95 % confidence level. To clarify, ‘lower limb asymmetry’ in the SG refers to the difference in values obtained for the lower limb corresponding to the convexity side of the spinal curve and the lower limb corresponding to the concavity side of the curve. Lower limb asymmetry in the CG refers to the difference in values obtained for the left and right lower limbs.

## Results

The data collected in this study was analysed at three levels: (1) Asymmetry in the SG against asymmetry in the CG, (2) Difference in magnitude of asymmetry between the SG and CG, and (3) Global mean values in the SG vs. CG. Table 2 and Table 3 present the results of this study.

**Table 2:** Intra-group analysis of results

			Study group			Control group		
			Cc	Cx	*p*	Left	Right	*p*
Temporal Parameters		Step Length (m)	0.625 ± 0.043	0.628 ± 0.044	0.47	0.653 ± 0.063	0.653 ± 0.056	0.99
		Step Speed (ms^−1^)	1.183 ± 0.139	1.199 ± 0.136	0.07	1.265 ± 0.116	1.271 ± 0.110	0.68
		Cadence (steps/min)	113.6 ± 10.6	114.7 ± 10.5	0.20	117.7 ± 10.5	118.0 ± 9.3	0.68
		Swing-to-Stance ratio	0.704 ± 0.069	0.696 ± 0.068	0.49	0.728 ± 0.083	0.727 ± 0.077	0.93
Vertical GRF values (% body weight)		Peak during Loading	103.5 ± 8.4	104.0 ± 9.4	0.57	102.2 ± 12.5	104.7 ± 11.4	0.13
		Peak during Propulsion	109.5 ± 8.0	110.4 ± 7.6	0.28	107.3 ± 11.6	110.2 ± 11.4	0.15
Peak EMG values (mV)		GL	0.442 ± 0.127	0.437 ± 0.141	0.80	0.570 ± 0.263	0.552 ± 0.246	0.63
		GM	0.588 ± 0.182	0.610 ± 0.205	0.54	0.711 ± 0.269	0.752 ± 0.332	0.41
		VM	0.211 ± 0.177	0.183 ± 0.123	0.34	0.188 ± 0.107	0.186 ± 0.099	0.93
Time (% Gait Cycle) at which the mean peak EMG value occurs	After Initial Contact	GL	42.4 ± 3.2	42.0 ± 2.8	0.42	40.7 ± 2.9	40.6 ± 3.2	0.80
		GM	39.3 ± 4.0	38.8 ± 4.6	0.52	37.9 ± 4.6	38.2 ± 3.1	0.66
		VM	12.0 ± 23.0	4.3 ± 3.6	0.07	13.8 ± 28.2	3.6 ± 2.0	0.10
	Before Toe Off	GL	16.4 ± 2.4	17.0 ± 2.6	0.08	17.3 ± 2.9	17.4 ± 2.9	0.82
		GM	19.5 ± 3.0	20.3 ± 4.3	0.33	20.1 ± 4.2	19.8 ± 2.2	0.71
		VM	53.2 ± 6.4	54.8 ± 4.5	0.15	52.9 ± 10.2	54.4 ± 3.4	0.51

Note: Side-to-side lower limb values are evaluated in terms of concavity (Cc) vs. convexity (Cx) side for the Study Group, and left vs. right for the Control Group

**Table 3:** Inter-group analysis of results

			Difference in Magnitude of Lower Limb Asymmetry			Global Mean		
			SG	CG	*p*	SG	CG	*p*
Temporal Parameters		Stride Length (m)	0.027 ± 0.032	0.030 ± 0.032	0.72	1.185 ± 0.124	1.280 ± 0.071	*0.002*
		Stride Time (s)	0.035 ± 0.026	0.036 ± 0.031	0.88	1.129 ± 0.135	1.022 ± 0.080	*0.001*
		Step Length (m)	0.013 ± 0.014	0.013 ± 0.013	0.96	0.626 ± 0.043	0.653 ± 0.059	0.06
		Speed of Gait (ms^−1^)	0.037 ± 0.033	0.051 ± 0.039	0.16	1.191 ± 0.135	1.268 ± 0.108	*0.03*
		Cadence (steps/min)	3.5 ± 3.2	3.6 ± 2.7	0.87	114.1 ± 10.3	117.8 ± 9.6	0.18
		Swing-to-Stance ratio	0.046 ± 0.042	0.032 ± 0.033	0.18	0.700 ± 0.061	0.728 ± 0.077	0.14
Vertical GRF values (% body weight)		Peak during Loading	3.7 ± 2.8	5.8 ± 5.9	0.09	103.8 ± 8.6	103.5 ± 11.5	0.90
		Peak during Propulsion	3.0 ± 3.7	6.0 ± 7.5	0.06	109.9 ± 7.5	108.8 ± 10.6	0.64
Peak EMG values (mV)		GL	0.089 ± 0.074	0.139 ± 0.120	0.06	n/a	n/a	n/a
		GM	0.139 ± 0.138	0.161 ± 0.172	0.62	n/a	n/a	n/a
		VM	0.097 ± 0.130	0.076 ± 0.061	0.46	n/a	n/a	n/a
Time (% Gait Cycle) at which the mean peak EMG value occurs	After Initial Contact	GL	2.1 ± 1.5	1.2 ± 1.0	*0.02*	42.2 ± 2.7	40.7 ± 2.9	*0.05*
		GM	2.5 ± 3.6	2.3 ± 2.4	0.81	39.0 ± 3.7	38.1 ± 3.5	0.34
		VM	8.4 ± 22.4	11.9 ± 28.0	0.61	8.1 ± 11.9	8.7 ± 13.9	0.88
	Before Toe Off	GL	1.5 ± 1.5	1.6 ± 1.2	0.90	16.7 ± 2.3	17.4 ± 2.7	0.34
		GM	2.6 ± 3.7	2.8 ± 2.5	0.87	19.9 ± 3.0	19.9 ± 2.8	0.95
		VM	3.7 ± 5.0	4.4 ± 9.7	0.74	54.0 ± 4.7	53.7 ± 5.4	0.80

The adopted speed of gait was found to be significantly slower in the SG compared to the CG. This is emphasised by the significantly shorter stride length and longer stride time of the SG. Furthermore, lower limb asymmetry with regards to the individual step speed in the SG tended towards being significantly different (*p* = 0.07): the mean speed of stepping with the lower limb corresponding to the concavity side of the spinal curve (1.18 ms^−1^) tended to be slower than the mean speed of stepping with the lower limb on the side of convexity (1.20 ms^−1^). However, intra- and inter-group differences in cadence and swing-to-stance ratio were not found to be statistically significant.

Peak vertical GRF values and peak EMG values of the selected muscles were not found to have significant intra- and inter-group differences; however, mean peak vertical GRF values were slightly higher in the SG than in the CG, especially during the Propulsion phase. Lower limb asymmetry with regards to time of onset of peak GL EMG values were significantly higher (*p* = 0.02) in the SG when compared to side-to-side asymmetry in the control subjects. Results indicate a significant increased level of dissimilarity within the SG with respect to side-to-side lower limb asymmetry which seems to be unrelated to the side of the scoliotic curve (*p* = 0.42). Furthermore, the onset of EMG peaks for the GL occur significantly later (*p* = 0.05) in the gait cycle of the SG than in the CG.

## Discussion

The primary objectives for this study were to analyse lower limb asymmetries of scoliosis subjects during level walking, and to analyse the differences in these lower limb asymmetries between the SG and the CG. Furthermore, inter-group differences in the selected variables were also analysed.

### Temporal parameters

The walking speed values for every subject participating in this study fall within the normal range (0.82-1.60 m/s ± 0.16) [22]. This indicates that, although the average walking speed was found to be lower in the SG, the values were not exceptionally low as to deviate from the norm. The lower average speed of gait in the SG could be the result of a decrease in either cadence or step length, or a combination of both [22]. No significant changes in cadence were revealed in this study. However, scoliotic subjects tended to have a shorter step length that the CG, while the stride length was significantly shorter. The shorter stride length and longer stride time both reflect the slower speed of gait in the SG. A diminished step length and a slower speed of gait in scoliotic subjects has been reported in the literature on more than one occasion, not necessarily in combination [9, 12, 13]. Furthermore, during intra-group analysis, results indicate that SG subjects tended to be faster when stepping with the limb corresponding to the covexity side of the curve, which may signify that stepping with that limb is more challenging. This corresponds to GRF results which are discussed in the next section.

Cadence and stride time are two closely related temporal parameters. The higher the stride time, the lower the cadence. If stride time is equal to 1 s, the cadence should then be equal to 120 steps/min. However, stepping with the convexity side was faster in the SG (Table 2). Hence, it follows that the stepping time is not symmetrical in the SG. This asymmetry is reflected in the mean stride time results (Table 3), and therefore, the relationship between cadence and stride time is not precisely exhibited for the SG in contrast to the CG.

Comparison of temporal parameters in intra-group analysis highlights the asymmetry in the SG, even though results are not statistically significant. Stride length is taken as the sum of the left and right step length. With reference to Table 2, there is a slight discrepancy in the step length and cadence of the SG – the step length on the concavity side was slightly shorter in relation to the opposite step. As a result of this asymmetry, the stride length is not double the step length for the SG in contrast to the CG since mean values are taken into consideration for inter-group analysis (Table 3).

In a comprehensive study by Mahaudens *et al.* [7], patient groups of both mild and severe scoliosis have slightly but significantly decreased step length compared to normal subjects when walking at a fixed speed (4 km/h). This decrease in step length was linked to the different pelvic orientation in scoliotic subjects [6, 9], which was in turn linked to the severity of the spinal deformity [6, 7, 9]. Further reinforcing this finding, it was discovered that step length increased by 4 % while cadence decreased by 2 % whist walking at a constant speed of 4 km/h following corrective surgical intervention of the spinal curve [14]. These results indicate that although spinal deformity occurs in the trunk, it influences the motion pattern of the lower limbs. Even in the absence of a spinal deformity, Thummerer *et al.* [23] concluded that walking speed has a significant association with spine and pelvic movements when studying the gait cycle of healthy young subjects (aged 1–16 years). Thus, the significantly lower self-selected speed of gait in the SG may be the result of the trunk movement asymmetry established in scoliosis [24]. Other possible justifications for the reduced average walking speed in subjects suffering from scoliosis include: decreased balance control [1, 25], increased energy cost of locomotion [8], decreased pulmonary efficiency [9] and resultant decreased efficiency of the gait cycle.

The swing-to-stance ratio is a useful clinical tool to detect gait deviations. Swing-to-stance ratio values obtained in this study lie within the range of 0.696-0.728, and thus fall outside the range of normal ratio values (0.63-0.64) [22]. A higher swing-to-stance ratio translates into a longer swing phase and a shorter stance phase, also impling an increased walking speed [22]. Thus, the lower swing-to-stance ratio observed in the SG indicates a slower speed of gait, which is a significant finding of this study. The lack of significant disparity between the swing-to-stance ratio of scoliotic subjects and non-scoliotic subjects is reinforced by comparable findings by Chen *et al.* [10]. In contrast, Mahaudens *et al.* [7] observed that the stance phase was slightly but significantly reduced in all scoliosis groups when compared to the norm. However, subjects participating in this study were obliged to walk at a constant speed of 4 km/h, forcing subjects to adjust their gait pattern to accommodate the selected speed, which may have resulted in an artificial gait pattern.

### Ground reaction force

This study only considers the vertical component of the GRF, in terms of body weight percentage, since it is the parameter of choice to characterise the dynamics behaviour of scoliosis subjects established in the literature [16,24]. Past research has suggested statistically significant differences in GRF between subjects with scoliosis and healthy persons [15]. Although such results were not reproduced in this study, mean peak vertical GRF values during both the Loading phase and the Propulsion phase were slightly higher in the SG than in the CG. The slower speed of gait adopted by scoliosis subjects reduces the momentum and as a consequence, the vertical acceleration [26, 27]. Thus, the higher vertical GRF points towards towards an inefficient gait pattern.

Herzog *et al.* [28] demonstrated that human gait is asymmetrical with respect to GRF, but claimed that symmetry between left and right lower limbs was greatest in the vertical forces which deviate by less than 4 %. On the other hand, Schizas *et al.* [15] have shown asymmetry of vertical GRF during gait to be more than 4 % in the scoliosis group, especially during loading/unloading, but were unable to relate it to the side or the extent of the spinal deformity. Although the presence of lower limb vertical GRF asymmetries during scoliotic gait is a logical assumption, such results are not reflected in this study. On the contrary, scoliotic subjects tended to be more symmetrical in this aspect than the norm, which is an interesting finding. Kramers-de Quervain *et al.* [24] also claim that vertical GRF asymmetries are not clinically relevant in scoliotic subjects since GRF values fall within the range recorded for healthy subjects by Herzog *et al.* [28].

Findings in the current study show that the mean vertical GRF peak values during both the Loading phase and the Propulsion phase were marginally higher for the lower limb corresponding to the convexity side of the scoliotic curve. In order to overcome inertia and allow motion of the lower limb, the vertical force must exceed the weight on that limb, which is higher on the convexity side. According to Bruyneel *et al.* [3, 16, 17], scoliosis subjects tend to bear extra weight on the lower limb corresponding to the convexity side of the spinal curve, making stepping with that limb more challenging. This raises the need for dynamic behaviour adjustment in order to maintain balance during gait and compensate for the spinal deformity. Gait initiation took systematically longer in right thoracic scoliosis subjects, than healthy subjects, though no significant differences in movement duration between left and right forward stepping were uncovered [16], as mirrored by results of this study. However, an increased GRF for the left lower limb (*i.e.* concavity side) during forward and lateral stepping was linked to the asymmetric pathology of scoliosis in the studies by Bruyneel *et al.* [3, 16, 17]. Interestingly, such findings conflict with the findings of the current study. Since subjects in the SG have different types of scoliosis at different levels of severity, while subjects in the studies by Bruyneel *et al.* [3, 16, 17] had right thoracic scoliosis, the results of the current study reflect a more global overview observation of vertical GRF asymmetry in the lower limbs. The lower limb asymmetries in the vertical GRF during gait are possibly dissimilar in scoliotic subjects with different characteristics of the scoliotic curve. This offers scope for further research.

### Electromyography

Dynamic electromyography (EMG) is an ideal gait analysis system used to classify the onset and relative intensity of muscle function through the measure of motor action potentials. This measurement is representative, but not equivalent to the muscle force [29], especially during gait. EMG profiles reflect the activity and consequently the function of each muscle during the gait cycle [30].

Ample research is available on trunk musculature [1, 8, 12, 18, 19, 31] in individuals with scoliosis. Trunk asymmetries lead to proprioception and mechanical dysfunction, which should be expressed in the gait pattern [1, 7, 15]. However, the literature on lower limb muscle activity in scoliosis subjects is limited. Apart from the restricted availability of literature, researchers find different ways to analyse EMG data, most frequently through normalisation. Since the aim of this study was to analyse peak EMG values for side-to-side lower limb asymmetries, normalisation was not required. However, this meant that inter-group comparison of peak EMG values was not possible.

This research has established that intra- and inter-group lower limb asymmetries in peak EMG values (mVolts) for the VM, GL and GM muscles are not significantly different. Nevertheless, there was some significance in differences in the time of onset of such EMG peaks. Syczewska *et al.* [12] recorded abnormal asymmetrical activity of the muscles along the vertebral column and the glutei muscles. On the other hand, Mahaudens *et al.* [7] documented a bilaterally increased EMG duration of Quadratus Lumborum, Erector Spinae, Gluteus Medius, and Semitendinosus muscles but computed no significant side-to-side asymmetry. Side-to-side asymmetry was also rejected by Chen *et al.* [10]. Furthermore, Le Blay *et al.* [32] evaluated muscle strengths with a dynamometer and discovered significant trunk and knee muscle weakness for people with scoliosis when compared to a control group.

VM muscle activity commences towards the end of the swing phase and rapidly increases to peak during the loading phase, at about 5 % of the gait cycle. Muscle effort reduces with the onset of the support phase and ceases by 15 % of the gait cycle [26]. For the SG, peak EMG values for the VM muscle on the side corresponding to the concavity of the curve occurred at 12.0 % of the gait cycle. The peak EMG value for the contralateral limb tended to occur earlier (4.3 %), indicating a delayed or prolonged loading phase for the lower limb corresponding to the concavity side of the scoliotic curve.

Gastrocnemius muscle activity has its onset in the early support phase, at approximately 10 % of the gait cycle, to provide support and stability of the ankle joint. Muscle activity progressively augments and reaches its peak at the end of the support phase (50 % of the gait cycle), for push off and forward propulsion, followed by a rapid decline during the end of the propulsion phase [26]. There were no significant findings in EMG characteristics of GM. However, the onset of EMG peaks for the GL occur significantly later (*p* = 0.05) in the gait cycle of the SG than in the CG. This indicates a delayed propulsion phase in scoliotic subjects. Lower limb asymmetry with regards to time of onset (after Initial Contact) of peak GL EMG values were significantly higher (*p* = 0.02) in the SG when compared to side-to-side asymmetry in the control subjects. This signifies that there is increased variation between lower limbs in the time of peak muscle activity of GL within the SG, when compared to the norm. Furthermore, there is side-to-side variation in the time of onset of GL EMG peaks within the SG, if considered in terms of the percentage of the gait cycle at the end of the propulsion phase before Toe Off. GL EMG peaks on the convexity side of the spinal deformity occur earlier in the gait cycle, when compared to the opposite limb within the SG. This finding was not statistically significant (*p* = 0.08) but is still noteworthy. Earlier activation of the GL on the convexity side may be an indication of prolonged muscle activation as required for the propulsion of the limb which bears extra weight. These results emphasise the presence of side-to-side variation in the time of onset and possibly duration of push off during the gait cycle.

### Limitations

Individuals who are aware of being under examination may alter their behaviour. However, participants were not informed which dynamic trials were being recorded. In addition, the location of the force plate was concealed hence subjects would not attempt to land their foot squarely on the forceplate. The magnitude of the curve is best determined by measurement of the Cobb angle [33], however for the purpose of this study, the spinal curvature was measured through surface asymmetry due to the lack of human, material and capital resources.

## Conclusions

The effects of scoliosis on the efficiency and energy requirements of locomotion are relatively high [9]. Mahaudens *et al.* [8] calculated that the pathological gait of scoliosis entailed a 30 % increase in energy requirements when compared to the norm. Scoliosis disrupts normal biomechanics [12] as indicated by the shorter stride length, longer stride time, slower speed of gait as well as the variation in time of peak muscle activation detected in scoliotic subjects when compared to the norm. These results of this study indicate a less efficient gait cycle, as underlined by the slower self-selected walking speed of scoliotic subjects. This slower speed of gait may be the result of multiple other factors, such as balance control and energy efficiency which could be the subject for further research.

## Notes

Presentation of this research:

This research was presented in the form of a poster presentation at the National Symposium of Health Sciences organised by the University of Malta, on the 24^th^ April 2014.
